# A realistic appraisal of boron neutron capture therapy as a cancer treatment modality

**DOI:** 10.1186/s40880-018-0280-5

**Published:** 2018-06-19

**Authors:** Rolf F. Barth, Zizhu Zhang, Tong Liu

**Affiliations:** 1 Department of Pathology, The Ohio State University, Columbus, OH 43210 USA; 2 Beijing Capture Technology Company, Ltd., Beijing, 102445 P. R. China

**Keywords:** Boron neutron capture therapy, Brain tumors, Head and neck cancer, Melanomas

## Abstract

Boron neutron capture therapy (BNCT) is a binary therapeutic modality based on the nuclear capture and fission reactions that occur when the stable isotope boron-10 is irradiated with neutrons to produce high-energy alpha particles and recoiling lithium-7 nuclei. In this Commentary we will focus on a number of papers that were presented at a Symposium entitled “Current Clinical Status of Boron Neutron Capture Therapy and Paths to the Future”, which was held in September 2017 at the China National Convention Center in Beijing. Results were presented by clinicians from Japan, Finland, the United States, the China mainland and Taiwan, China who have been working in the multiple disciplines that are required for carrying out clinical BNCT. The main focus was on the treatment of patients with malignant brain tumors, recurrent tumors of the head and neck region, and cutaneous melanomas. The results obtained in treating these patients were reported in detail and, although most of the patients with brain tumors and head and neck cancer were not cured, there was evidence of some clinical efficacy. Although there are a number of problems that must be addressed, further clinical studies to evaluate the efficacy of BNCT are warranted. First, despite considerable effort by numerous investigators over the past 40 years, there still are only two boron-containing drugs in clinical use, l-boronophenylalanine (BPA) and sodium borocaptate (BSH). Therefore, until new and more effective boron delivery agents are developed, efforts should be directed to improving the dosing and delivery of BPA and BSH. Second, due to a variety of reasons, nuclear reactor-based BNCT has ended except for its use in the China mainland and Taiwan. Therefore, the future of BNCT depends upon the results of the ongoing Phase II clinical trials that are being carried out in Japan and the soon to be initiated trials that will be carried out in Finland. If the results obtained from these clinical trials are sufficiently promising, then BNCT will have a clear path to the future, especially for patients with the therapeutically challenging malignancies that in the past have been treated with reactor-based BNCT.

## Background

In September 2017 a Symposium entitled “Current Clinical Status of Boron Neutron Capture Therapy and Paths to the Future” was held at the China National Convention Center in Beijing. This symposium brought together a group of clinicians and scientists from Japan, Finland, the United States, and the China mainland and Taiwan, China who have been working in the multiple disciplines that are required for carrying out clinical Boron Neutron Capture Therapy (BNCT). A total of 14 presentations were given at the Symposium, and this Commentary will focus on some of the major issues raised by them, including three reports that accompany this Commentary.

**Fig. 1:**
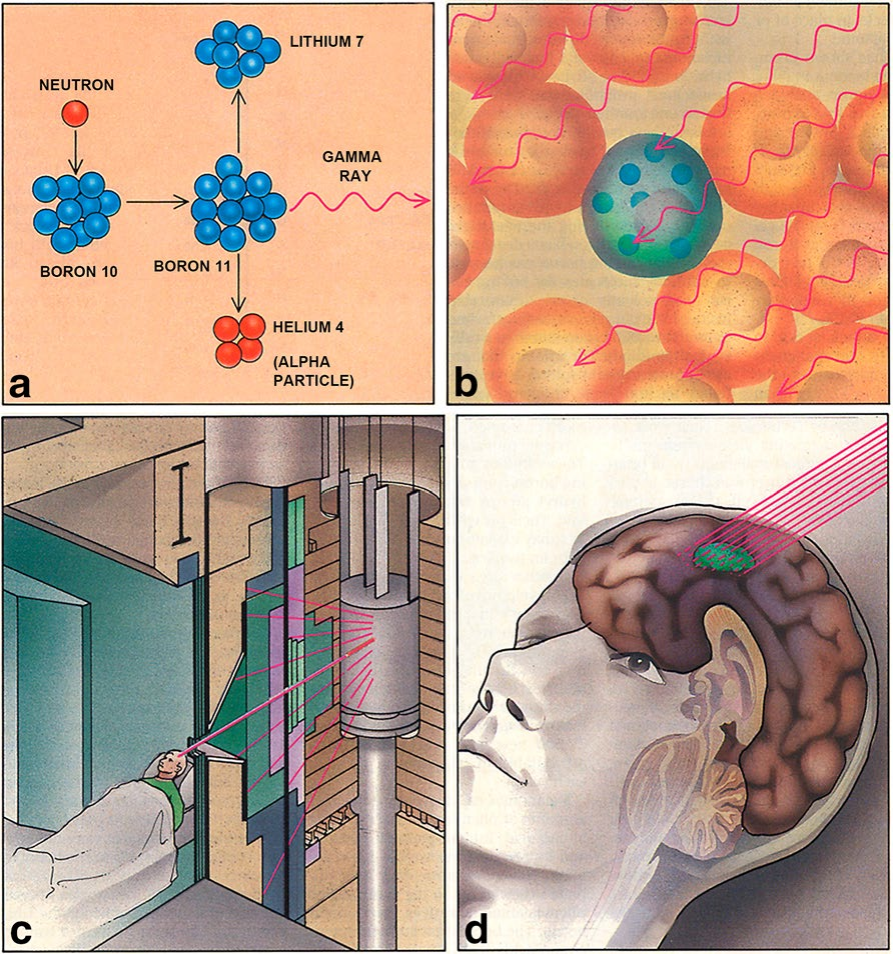
BNCT is based on the nuclear capture and fission reactions that occur when boron-10, a nonradioactive constituent of natural elemental boron, is irradiated with low-energy (0.025 eV) thermal neutrons or, alternatively, higher-energy (10,000 eV) epithermal neutrons, which lose energy as they penetrate tissues and become thermalized. This capture reaction results in the production of high linear energy transfer (LET) alpha particles (^4^He) and recoiling lithium-7 (^7^Li) nuclei (Fig. 1a). In order to be successful, a sufficient amount of ^10^B must be selectively delivered to the tumor (~ 20–50 µg/g or ~ 10^9^ atoms/cell) (Fig. 1b) and a collimated beam of neutrons (Fig. 1c) must be absorbed by the tumor (Fig. 1d) to sustain lethal damage from the ^10^B(n,α)^7^Li capture reaction. The destructive effects of the alpha particles are limited to boron-containing cells and, because they have very short pathlengths in tissues (5–9 µm), BNCT provides a way to selectively destroy malignant cells and spare surrounding normal tissue, making it, in theory, an ideal type of radiation therapy

BNCT is based on the nuclear capture and fission reactions that occur when boron-10, a non-radioactive constituent of natural elemental boron, is irradiated with low-energy (0.025 eV) thermal neutrons or alternatively, higher-energy (10,000 eV) epithermal neutrons, which lose energy as they penetrate tissues and become thermalized [1]. This capture reaction results in the production of high linear energy transfer (LET) alpha particles (^4^He) and recoiling lithium-7 (^7^Li) nuclei (Fig. 1a). In order to be successful, a sufficient amount of ^10^B must be selectively delivered to the tumor (~ 20–50 μg/g or ~ 10^9^ atoms/cell) (Fig. 1b), and a collimated beam of neutrons (Fig. 1c) must be absorbed by the tumor (Fig. 1d) to sustain a lethal ^10^B(n, α)^7^Li capture reaction. The destructive effects of the alpha particles are limited to boron containing cells and since they have very short pathlengths in tissues (5–9 μm), in theory BNCT provides a way to selectively destroy malignant cells and spare surrounding normal tissue, making it an ideal type of radiation therapy.

Despite the work of numerous investigators described in a number of reviews [2–5], the most recent of which appears in this issue of the journal [6], there are only two drugs that have been used clinically as boron delivery agents for neutron capture therapy. The first is a polyhedral borane anion known as sodium borocaptate (BSH) and the second is a dihydroxyboryl derivative of phenylanine known as boronophenylalanine (BPA). It is not for a lack of trying that no other boron delivery agents have been developed, but only these two have been sufficiently promising to warrant clinical biodistribution and therapy studies in humans.

## Overview of clinical studies

Clinical interest in BNCT has focused primarily on high grade gliomas [7–10], and more recently on patients with recurrent tumors of the head and neck (HN) region [11–18] who have failed conventional therapy. BNCT is a biologically rather than a physically targeted type of radiation therapy, and therefore it theoretically should be possible to selectively destroy tumor cells dispersed in normal tissue, *providing* that sufficient amounts of ^10^B and thermal neutrons are delivered to the individual tumor cells. In this Commentary we will provide a brief update on BNCT as it relates to the treatment of high grade gliomas, recurrent cancers of the head and neck region and melanomas, as reported by clinicians who presented at the Symposium. We also will discuss some of the challenges that must be addressed in order for BNCT to transition from an experimental modality to one that is more widely accepted clinically. Up until 2014 the source of neutrons used clinically for BNCT were especially dedicated nuclear reactors that produced either thermal or epithermal neutrons. However, since then three Japanese companies, Sumitomo Heavy Industries, Hitachi, and Mitsubishi, and one American company, Neutron Therapeutics (Danvers, Massachusetts, USA), have manufactured accelerator neutron sources that can be sited in hospitals and produce epithermal neutron beams. Three of these accelerators currently are being evaluated in Phase II clinical trials in Japan to assess their safety and efficacy. The fourth one, manufactured by Neutron Therapeutics, produces neutrons from a ^7^Li target with 2.6 MeV protons at 30 mA current. It will enter into clinical use in Finland in the latter part of 2018 [19]. The Japanese Phase II trials are nearing completion and it is anticipated that the clinical results will be reported sometime in the latter part of 2018 or early 2019.

## Clinical studies on BNCT of high grade gliomas and head and neck cancers

Three papers presented at the Symposium reported on studies describing the clinical results obtained using BNCT to treat patients with malignant brain tumors, the largest number of which had high grade gliomas. Koji Ono, who has been the driving force in the Japanese BNCT program, presented a review of what has been accomplished to date and what needs to be done in the future to advance BNCT. Since, for all intents and purposes, nuclear reactor-based BNCT probably has ended in Japan, the future rests with accelerator-based neutron sources, as indicated above. Miyatake et al. [9, 10] and Kawabata et al. [20] at Osaka Medical College [9, 10, 20] have treated the largest number of brain tumor patients, followed by the Finnish group under the leadership of Joensuu and Kankaaranta [21, 22], and finally a small number of patients treated by Chen in Taiwan [23]. In all of these studies, L-BPA as a fructose complex (BPA-F) was used as the delivery agent. Miyatake et al. [9, 10] initiated their studies on the treatment of brain tumors in 2002 and since then they have treated 58 patients with newly diagnosed high grade gliomas, 50 with recurrent tumors, and 32 patients with recurrent high grade meningiomas [24], for a total of 140 patients with 167 applications of BNCT. Their results have been reported in detail elsewhere [9, 10, 20] and are briefly summarized as follows. Focusing on the 50 patients with recurrent high grade gliomas, the combination of L-BPA and BSH was used as the boron delivery agents, preceded in most patients by positron emission tomography (PET) imaging using ^18^F-BPA to determine the uptake of BPA-F prior to carrying out BNCT. Two-thirds (8 of 12) of the patients, who had contrast-enhanced regions by magnetic resonance imaging (MRI), showed a decrease in their tumor size during the follow-up period. In a cohort of 22 patients with recurrent gliomas there was a significant prolongation in mean survival time of 9.1 months following BNCT versus 4.4 months for those that had received other types of salvage therapy following their recurrence. However, 30 of the 87 patients who were treated between January 2002 and July 2013 subsequently developed cerebrospinal fluid (CSF) dissemination of their tumors, and a disproportionate number of these had small cell glioblastomas (GBM) [25]. Beginning in 2012 a Phase II clinical trial was initiated using a cyclotron-based accelerator neutron source manufactured by Sumitomo Heavy Industries, however, the results of this trial have yet to be reported. Finally, Chen [23] presented a preliminary report on a group of six patients with recurrent gliomas who were treated at the Taipei Veterans General Hospital in 2017, but no definitive statements relating to their clinical results were made at the time of his presentation.

## Clinical studies in Finland

A total of 249 patients have been treated between 1999 and 2012 at the FiR 1 research reactor in Helsinki, Finland with more than 300 applications of BNCT. These patients received L-BPA-F as the boron delivery agent. A majority of them had either primary (n = 39) or recurrent (n = 58) high grade gliomas [21, 22] or cancers of the HN region (n = 140) [11, 12]. Two clinical trials for HN cancers were carried out, one with BNCT alone (n = 30) and the other in combination with the epidermal growth factor receptor (EGFR)-targeting monoclonal antibody cetuximab (n = 19) [12]. A group of 71 patients were treated outside of the clinical trials, and 4 patients with inoperable tumors were treated with BNCT, followed by chemotherapy and photon irradiation [11]. Patients with other tumor types that have been treated included three with melanomas, seven with meningiomas, and one with a lymphoma.

The dose of BPA-F was 400 mg/kg infused over 2 h [22]. Patients with primary GBMs had median survival times (MSTs) ranging from 11.0 to 21.9 months. Those patients with recurrent tumors, who had been previously treated with BNCT within the clinical trial (n = 22), had a MST of 7.3 months [22, 26], and those patients who received BPA-F at a dose > 290 mg/kg survived longer [22]. It is noteworthy that those patients with recurrent gliomas, who had received BNCT followed by conventional photon beam irradiation, tolerated it well. Adverse effects were usually grade 1 or 2 (mild or moderate) in severity. Seizures were the most frequent grade 3 complication and occurred in 18% of these patients, and none were life-threatening (grade 4). The response rates of the 30 patients with HN cancer treated with BNCT were better than those seen in patients with gliomas. Forty-three percent achieved a complete response, 30% had partial responses, and 20% had stable disease for a median of 8.5 months and 3% progressed [12]. The MST of the HN cancer patients was 13.0 months and the 2 and 4 year survival rates were 30% and 18%, respectively. The median duration of local control, defined as no recurrent tumor at the site of the primary, was 7.9 months and the 2 and 4 year control rates were 27% and 16%, respectively. Mucositis (54% of patients) and oral pain (54%) were the most common acute grade 3 adverse event, followed by fatigue (32%). One patient with newly diagnosed, inoperable HN cancer, who first had received BNCT followed by chemotherapy and photon irradiation, achieved a complete durable response [11].

Reactor-based BNCT ended in Finland in 2012 due to financial issues relating to the operation of the FiR1 research reactor. In 2018, an electrostatic accelerator-based neutron source, designed and fabricated by Neutron Therapeutics Inc., is being installed at Helsinki University Central Hospital, and clinical trials involving patients with recurrent HN cancer will be initiated once approval has been given by the Finnish health authorities. Based on the results obtained with this accelerator neutron source, its use will be extended to other types of cancer.

## Challenges in treating gliomas with BNCT

High grade gliomas are among the most difficult human malignancies to treat. The clinical results obtained by Miyatake et al. [9, 10] and Kawabata et al. [20] and the Finnish patients treated by Kankaanranta et al. [21, 22], and reported by Koivunoro at the Symposium, still have not gained wide acceptance of BNCT as a cancer treatment modality. This is hardly surprising since the single greatest advance in the treatment of patients with high grade gliomas has been the combination of post-surgical photon irradiation with the concomitant administration of temozolomide (TMZ) followed by repetitive cycles of TMZ, which resulted in a modest increase in median overall survival. This regimen was based on a study carried out by the European Organization for the Research and Treatment of Cancer (EORTC) [27, 28] consisting of 579 patients randomized to two arms, undergoing surgery plus either photon radiation alone or photon radiation in combination with TMZ, in order to demonstrate an increase in median overall survival of 2.5 months, which statistically was highly significant [28]. Therefore, barring either some major breakthrough in the development of new brain tumor-localizing boron delivery agents or a large, randomized clinical BNCT trial, it probably will be difficult to obtain data that will convince a broad audience of clinicians who treat patients with high grade gliomas that BNCT has much to offer other than a type of salvage therapy for those patients with recurrent tumors who have been treated to tolerance and have no other treatment options. Short of developing new and more effective boron delivery agents for BNCT of brain tumors, the best hope for enhancing its clinical efficacy would be to improve the dosing paradigm by increasing the dose of BPA and the infusion time, as has been reported by the Swedish group [29–31], or the use of novel physical methods to enhance the delivery of BPA and BSH, such as pulsed ultrasound (US) [32–34]. The use of pulsed US, which has been shown to transiently disrupt the blood–brain barrier (BBB), is one such approach that could improve not only the uptake of BPA and BSH but also their microdistribution within the tumor.

## Treatment of recurrent tumors of the head and neck region with BNCT

The second largest group of patients who have been treated by BNCT are those with recurrent tumors of the HN region who have had surgery, followed by chemotherapy and photon radiation with doses that have reached normal tissue tolerance levels and for whom there are no other treatment options. Although the total number of patients treated in Japan, Finland, and Taiwan are relatively small, there have been some very impressive clinical results [1, 11–18, 35]. Wang et al. [36] presented his results at the Symposium and they are briefly summarized as follows. A total of 17 patients with recurrent HN tumors, all of whom had multi-modality standard therapy, received BNCT using BPA-F as the boron delivery agent with two administrations of BNCT at 28-day intervals. Although the response rate was high (12 of 17 patients) and toxicity was acceptable, recurrence within or near the treatment site was common. This also has been the experience of Japanese and Finnish clinicians who also have treated patients with recurrent HN tumors. The basic problem resulting in recurrence following BNCT most likely has been due to non-homogeneous uptake of BPA-F with poor microdistribution in some regions of the tumor. Short of the development of new boron delivery agents, the best hope for improving the response and cure rates would be to optimize the dosing paradigm and delivery of BPA, either alone or in combination with BSH, which has not, as yet, been evaluated. Here, biodistribution studies using ^18^F-BPA PET and pretreatment biopsies of different parts of the recurrent tumor could be very useful, not only for treatment planning but also for improving the therapeutic results. In contrast to patients with high grade gliomas, a randomized clinical trial should be possible in Taiwan or Finland, which have large numbers of patients with recurrent HN cancer.

## Challenges relating to the use of BPA and BSH as boron delivery agents

The optimum dosing paradigm and delivery of BPA either alone or in combination with BSH in patients with high grade gliomas has yet to be determined. As reported by the Swedish group [29–31], increasing the dose of BPA and the duration of the infusion time would be a good starting point, but improving tumor uptake and microdistribution could require more than this. Again, short of developing new and more effective boron delivery agents, better ways to enhance tumor uptake and the microdistribution of BPA should be explored. One possible approach would be to use pulse-focused US to enhance its delivery for patients with either gliomas or HN cancer [37–39]. As described by Wood and Sehgal [34] in a recently published review, the delivery of chemotherapeutic agents has been studied using US alone or in combination with the administration of drug-loaded microbubbles. Two experimental studies in mice specifically relevant to HN cancer have been reported. In the first study [37] the luciferase-positive HN cancer cell line SCC1 was implanted subcutaneously into the flanks of nude mice. Microbubbles triggered by localized US enhanced the delivery of cetuximab labeled with a near-infrared dye. Optical imaging and direct measurements revealed that US resulted in a significant increase in cetuximab delivery, and tumor size at 24 days following implantation was significantly less in treated mice versus untreated control mice. More directly relevant to BNCT, Wu et al. [40] have employed high-intensity focused ultra-sound (HIFU) to enhance the uptake of BPA-F in nude mice bearing intra-oral xenografts of a human squamous cell carcinoma cell line designated SASC03. In vivo PET imaging studies using ^18^F-BPA-F revealed enhanced tumor uptake with no concomitant increase in normal tissue uptake. These two studies suggest that pulsed US should be evaluated clinically as a possible way to enhance the uptake and microdistribution of BPA-F in patients with HN cancer who are potential candidates for treatment by means of BNCT.

## Treatment of cutaneous melanomas with BNCT

Based on the pioneering studies of Mishima et al. [41–43], the third category of tumors that were discussed at the Symposium focused on melanomas, and two papers were presented. The first was by Zhang et al. [44] on the treatment of three Chinese patients with cutaneous melanomas using a compact In-Hospital Neutron Irradiator (IHNI), specially designed and fabricated for BNCT [44]. One of the three was a patient with an acral melanoma on the sole of his foot who had declined surgery. As reported by Yong et al. [45], there was complete eradication of the tumor, as determined by a biopsy at 9 months and PET imaging with ^18^F-glucose at 24 months. Two other patients, one with an acral lentiginous subungual melanoma of the right thumb and the other with multiple metastatic cutaneous nodules on the right leg, showed partial responses [45]. Hiratsuka presented a summary of the Japanese clinical results using BNCT to treat patients with cutaneous melanomas [41–43]. As summarized by Fukuda et al. [46], 32 patients (11 men and 21 women) with cutaneous melanoma who ranged in age from 50 to 85 years at the time of treatment, were treated with BNCT between July 1987 and June 2014 using BPA-F as the boron delivery agent. The overall complete regression (CR) rate was 78% (25/32) with 81% (22/27) for primary and 60% (3/5) for metastatic lesions. Among the patients with primary lesions, the CR rates were 33% (1/3) for nodular melanomas (NM) and 87.5% (21/24) for non-nodular melanomas. The complications most frequently observed were edema and cutaneous erosion at the site of irradiation. Overall, 28 of 32 patients had mild acute responses, 4 patients had moderate or severe cutaneous erosions that required medical intervention, and two of them had grade 4 toxicities consisting of soft tissue necrosis that required surgical excision and skin grafting. In summary, favorable clinical responses were obtained for the treatment of primary cutaneous melanomas with the exception of nodular melanomas. Since melanomas have a high propensity to metastasize, the possible combination of BNCT with new immunotherapeutic approaches [47, 48] would provide a better rationale to treat melanomas in difficult anatomic regions, such as the vulva, with BNCT [49].

## Treatment of genital cancers with BNCT

As described in a review in the same issue of the journal as this Commentary, Hiratsuka et al. [49] have used BNCT to treat one woman with a melanoma of the vulva, a second with extramammary Paget’s disease (EMPD) of the vulva and labia, and two men with EMPD of the scrotum and penis or scrotum and perianal area. Briefly summarized, BPA-F was administered intravenously over 2 h and this was followed by neutron irradiation. The minimum dose for tumor control was assumed to be either 20 Gy-Eq for EMPD or 25 Gy-Eq for the melanoma. There were striking clinical responses and all of the lesions regressed completely within 6 months, and there were no recurrences in the radiation field during the follow-up periods ranging from 1.6 to 6.9 years. Although both melanoma of the vulva and EMPD of it and the penis are relatively rare malignancies, these tumors unfortunately are very difficult to treat since the surgery can be very mutilating and the tumors are poorly responsive to conventional photon irradiation. Clearly, a larger number of patients need to be treated before any definitive statements can be made, but these results suggest that BNCT may be a very promising treatment for these malignancies. Although the incidence of these tumors is very low, in a country such as China with a population in excess of 1.3 billion, there could be a very large number of patients who might be considered as candidates for treatment by means of BNCT, especially in the case of melanoma of the vulva, when combined with immunotherapy, which recently has been shown to be very effective in treating patients with metastatic melanoma who have failed all other treatments [47, 48]. BNCT for EMPD of the penis and scrotum, combined with anti-PD1 immunotherapy, may represent a significant clinical advance in the treatment of this malignancy.

## Conclusions

In this Commentary we have summarized the current clinical experience using BNCT to treat patients with brain tumors, recurrent tumors of the head and neck region, and cutaneous and extracutaneous melanomas and EMPD. The clinical results obtained in treating patients with genital melanoma and EMPD are, in and of themselves, quite impressive. This would be a very promising group of patients to enroll in an expanded clinical trial. The challenges in treating patients with high grade gliomas by means of BNCT are significant but some new approaches, discussed in this Commentary, would be a reasonable path to follow until that time one or more new boron delivery agents reach the point of clinical evaluation. Finally, although there have been some striking clinical responses using BNCT to treat patients with HN cancer, many of these patients have had recurrences at the site of irradiation. Several suggestions have been made as to how to achieve better tumor uptake and microdistribution of BPA, and these could be carried out even in the absence of any new boron delivery agents. In conclusion, BNCT still remains an attractive twenty first century treatment option for hard to treat types of human cancers, but the problems associated with this modality, including the lack of new and better boron delivery agents [50], the uncertainty regarding accelerator neutron sources, and imprecise radiation dosimetry, must be surmounted if it ever will become anything more than a seductively attractive but unrealistic therapeutic modality.

## Abbreviations

ABNS: accelerator based neutron sources
BBB: blood–brain barrier
BNCT: boron neutron capture therapy
BPA: boronophenylalanine
BPA-F: boronophenylalanine-fructose
BSH: sodium borocaptate
CR: complete regression
CSF: cerebrospinal fluid
EGFR: epidermal growth factor receptor
EMPD: extramammary Paget’s disease
GBM: glioblastoma
HIFU: high-intensity focused ultrasound
HN: head and neck
MRI: magnetic resonance imaging
MST: median survival time
NM: nodular melanomas
PET: positron emission tomography
US: ultrasound
